# Management of generalized eruptive keratoacanthomas: A case report and literature review

**DOI:** 10.1016/j.jdcr.2024.01.037

**Published:** 2024-02-29

**Authors:** Kristiana Marie Jordan, Sarthak Saxena, Alyssa Ashbaugh Ortega, Maija Kiuru, Danielle Tartar

**Affiliations:** a University of California, Davis, School of Medicine, Sacramento, California; b Department of Dermatology, University of California, Davis, Sacramento, California

**Keywords:** GEKA, generalized eruptive keratoacanthomas, KA, keratoacanthoma

## Introduction

Generalized eruptive keratoacanthomas (GEKA) is a distinctive and uncommon variant of keratoacanthoma, presenting with hundreds of small skin-colored to erythematous, umbilicated keratotic papules.^1^^,^^2^ While the etiology of GEKA is unknown, associations have been made with environmental factors such as exposure to ultraviolet radiation, immunological abnormalities, viral exposures, trauma, and immunomodulatory medications. Systemic treatment is often most practical given the widespread distribution of GEKA; however, results are variable and often associated with adverse effects.^3^

GEKA poses treatment challenges, primarily due to its rapid progression and lack of standardized treatment approach. Accurate diagnosis and timely intervention are crucial, as misdiagnosis or delayed treatment can lead to complications and unnecessary patient distress. Here, we present the management approach to a patient with GEKA as well as a brief literature review regarding trends in management of GEKA.

## Case

**Fig 1:**
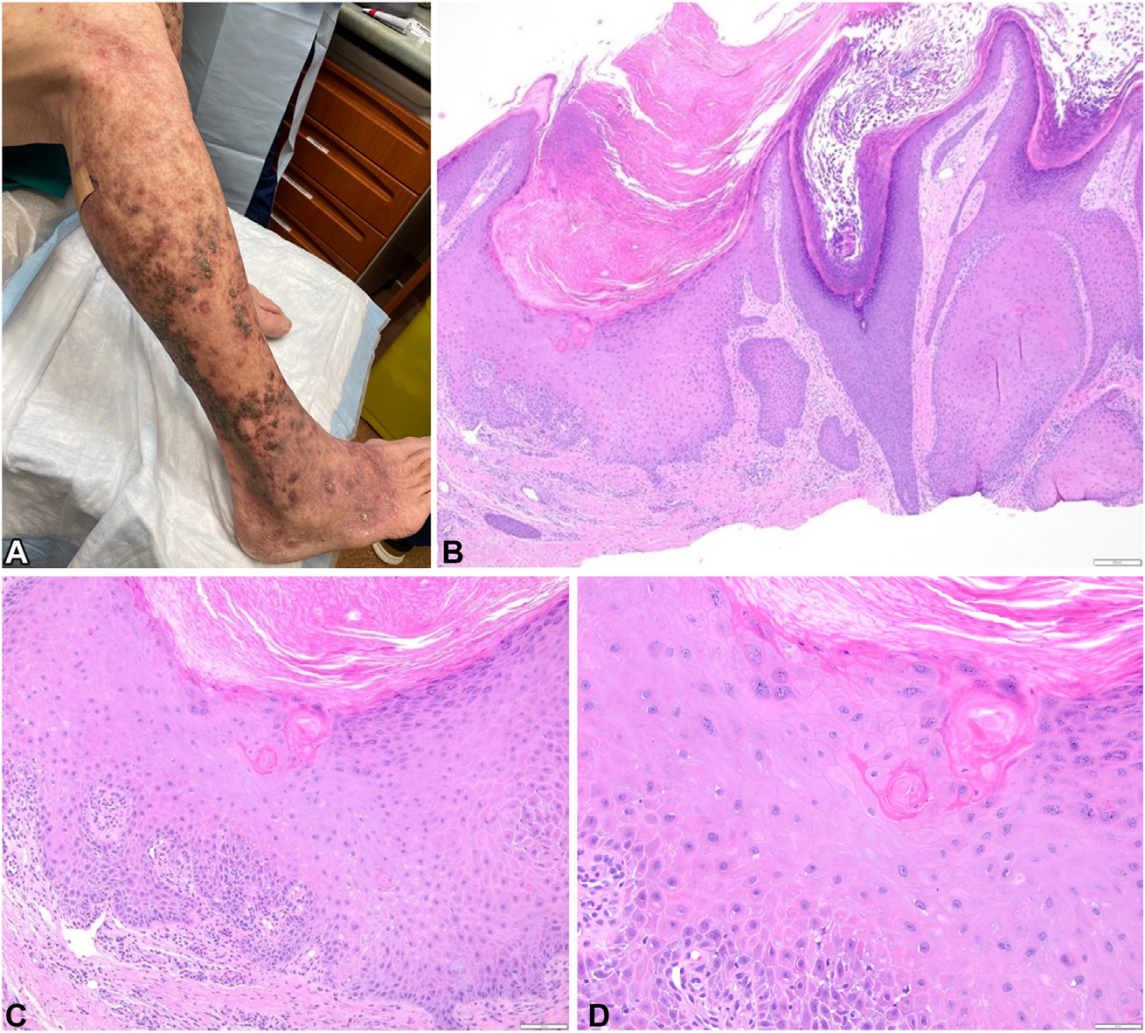
Generalized eruptive keratoacanthomas before treatment. **A,** Clinical presentation of generalized eruptive keratoacanthomas with secondary retention hyperkeratosis on the right distal extremity prior to treatment. Histopathology from a shave biopsy of generalized eruptive keratoacanthomas on the left distal pretibial region at (**B**) 4×, (**C**) 10×, and (**D**) 20× demonstrating a verrucous endophytic epithelial proliferation containing large keratinocytes with abundant pale eosinophilic cytoplasm and peripheral atypia.

A 75-year-old male presented with a 6-month history of persistent, pruritic bumps on the arms, shins, thighs, feet, and back. On physical exam, numerous 5 to 15 mm erythematous crateriform papules were present on the trunk and extremities, accompanied by pronounced erythema on the shins bilaterally (Fig 1, *A*). There was no mucosal involvement. Biopsies revealed multiple keratoacanthomas (GEKA; Fig 1, *B-D*). He was initially treated with higher dose acitretin (titrated to 50 mg daily), which lead to an elevation in liver enzymes.

The patient was transitioned to the epidermal growth factor receptor inhibitor erlotinib 150 mg daily by his oncology team but developed a pruritic acneiform dermatitis on the face and trunk within 1 week. He continued erlotinib for a 3-month trial, as recommended by his medical oncologist, but due to both rash and lack of clinical response, erlotinib was discontinued with resolution of the dermatitis.

The patient was next treated with the programmed cell death protein-1 inhibitor cemiplimab which resulted in a rapidly progressive, severe lichenoid eruption affecting large areas of the trunk and extremities. Biopsies of the left proximal pretibial region and left ventral proximal forearm were consistent with a lichenoid drug reaction. Due to the severity of his pruritus, cemiplimab was held, and the patient was prescribed a slow prednisone taper (beginning at 1 mg/kg) over 1.5 months as well as triamcinolone 0.1% ointment, with ultimate resolution of the lichenoid eruption.

**Fig 2:**
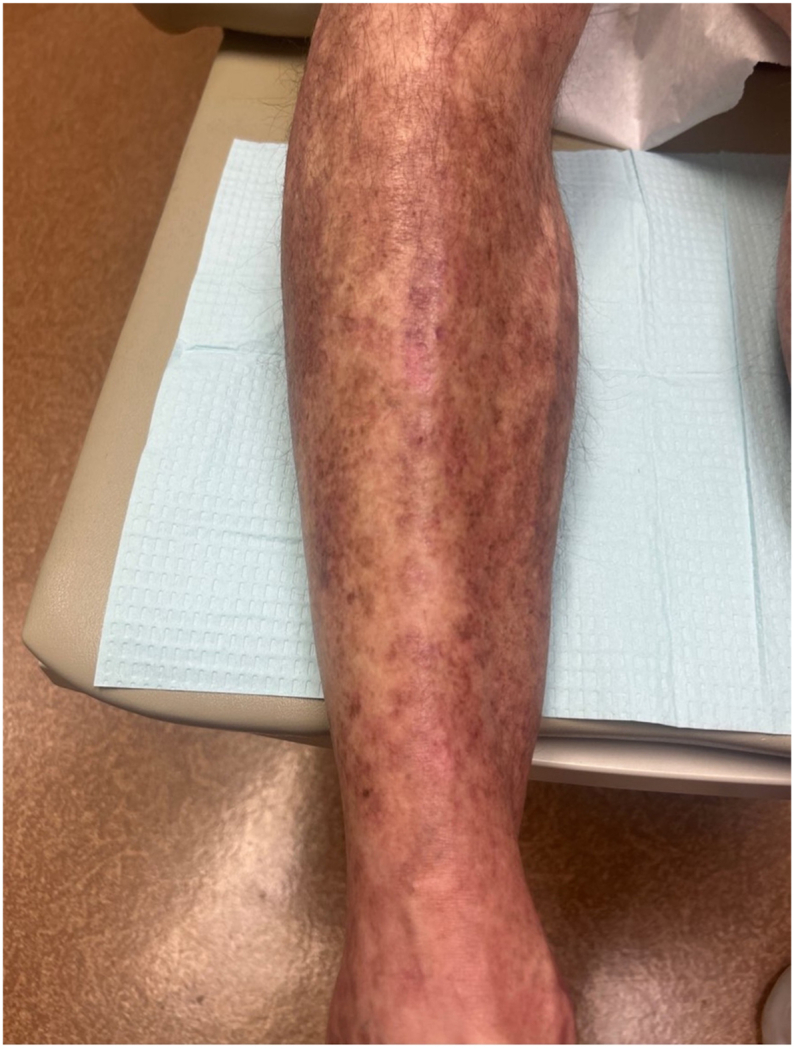
Generalized eruptive keratoacanthomas after treatment and resolution of lichenoid drug reaction. Right distal extremity showing resolution of generalized eruptive keratoacanthomas with associated postinflammatory hyperpigmentation changes after treatment.

Though the lichenoid drug reaction had resolved, his GEKA remained untreated. The patient was next treated with topical 5-fluorouracil weekly with unna boots; however, did not tolerate this secondary to pain. Intralesional triamcinolone (40 mg/mL) had no effect on GEKA lesions. Oral methotrexate (MTX) was not used given the patient’s prior history of transaminitis and the patient declined intralesional MTX. It was then decided to reinitiate treatment with oral acitretin at 10 mg daily and perform therapeutic shave removals of the most symptomatic lesions. Acitretin 10 mg daily was tolerated well without side effects. The patient returned to clinic 1 month later with decreased burden of keratotic papules and nodules on the lower extremities. Upon continuing this regimen of acitretin 10 mg daily for an additional 4 months, the patient experienced significantly improved disease burden, with only few remaining keratotic papules and nodules of the bilateral lower extremities noted at each visit (Fig 2). He is now on a maintenance dose of acitretin 10 mg every other day and very satisfied with his improvement.

## Discussion

Forty-one cases of GEKA have been published worldwide from 2018 to 2023, each with unique provoking factors (Table I). Common offenders are checkpoint inhibitor therapeutics—namely nivolumab and pembrolizumab. Other etiologies have included trauma, tattoos, 5-fluorouracil, and idiopathic causes. Due to the variety of etiologies and stubborn course of disease, multiple treatments have been utilized, yielding inconsistent results, and varied adverse events.^4^

**Table I:** GEKA cases that have been published worldwide from 2018 to 2023

Anatomic locations	Etiology (Weeks to onset)	Age range	Attempted treatments	Successful treatments	References
Face	PM8001 [PDL1 & TGF-β Inhibitor] (6)	67	d/c PM8001	d/c PM8001	Qi et al^6^
Trunk	s/p excision	84	Intralesional MTX	Intralesional MTX	Gualdi et al^12^
Extremities	Dupilumab (6)	33-92	Excision Topical 5-FU	Excision Topical 5-FU	Patchinsky et al^7^
	Pembrolizumab (15, 22, 40) & prednisolone		Acitretin Nicotinamide	Acitretin Nicotinamide	Star et al^10^
	Pembrolizumab (28, 61) & prednisolone		Excision Nicotinamide Topical steroids Decreased pembrolizumab frequency	Excision Nicotinamide Topical steroids Decreased pembrolizumab frequency	
	Pembrolizumab & ipilimumab (8, 14, 20, 22, 50)		Excision Acitretin Nicotinamide	Excision Acitretin Nicotinamide	
	Pembrolizumab with either epacadostat or placebo (10, 42)		Excision Oral & topical steroids	Excision Oral & topical steroids	
	Pembrolizumab (15)		Topical steroids Oral steroids Acitretin	Acitretin	Schwartz et al^11^
	Pembrolizumab & ipilimumab (8)				
	Pembrolizumab & ipilimumab (16)				
	Nivolumab (18)				
	Nivolumab (19)				
	Pembrolizumab (12)				
	Nivolumab (51)				
	Dupilumab (4)		Excision	Excision Spontaneous regression	Gleeson et al^13^
	Moderna mRNA-1273 Covid-19 vaccination		Mohs Intralesional 5-FU	Mohs Intralesional 5-FU	Ahmed et al^14^
	Topical 5-FU (2)		Acitretin Excision Intralesional 5-FU	Acitretin Excision Intralesional 5-FU	Cruzval-O’Reilly et al^16^
	Spironolactone (3)		Oral MTX Excision	Oral MTX Excision	Kunadia et al^18^
	Idiopathic		Excision Topical imiquimod Lapacho tea dressings Topical 5-FU	Excision Topical imiquimod Lapacho tea dressings Topical 5-FU	Havenith et al^19^
	Tattoo		Excision Topical tazarotene Cryotherapy	Excision Topical tazarotene Cryotherapy	Giberson et al^20^
	Idiopathic		Acitretin	Acitretin	Relvas et al^21^
	Tattoo (2)		Intralesional MTX	Spontaneous regression Intralesional MTX	Linares-Gonzales et al^22^
	Laser tattoo removal (1)		Intralesional triamcinolone	Spontaneous regression Intralesional triamcinolone	Hoss et al^23^
	Idiopathic		Excision Intralesional 5-FU Acitretin Oral nicotinamide Intralesional MTX Topical 5-FU PDT Intralesional triamcinolone	Excision Intralesional 5-FU Acitretin Oral nicotinamide Intralesional MTX Topical 5-FU PDT Intralesional triamcinolone	Marka et al^25^
	Nivolumab		Weekly wound care with antibacterial wound dressing, conforming stretch bandages, and thromboembolism deterrent hose Weekly occlusion and compression with zinc oxide gauze bandage (unna boot)	Weekly wound care with antibacterial wound dressing, conforming stretch bandages, and thromboembolism deterrent hose Weekly occlusion and compression with zinc oxide gauze bandage (unna boot)	Crow et al^26^
	Idiopathic		Excision Intralesional 5-FU	Intralesional 5-FU	Seger et al^27^
	Idiopathic		Mohs Acitretin	Mohs Acitretin	Dyson et al^28^
	Nivolumab (4)		None	Spontaneous regression	Fujimura et al^31^
	Nivolumab		ED&C Spontaneous regression after d/c nivolumab	ED&C Spontaneous regression after d/c nivolumab	Antonov et al^33^
	Nivolumab		Topical clobetasol Intralesional triamcinolone	Topical clobetasol Intralesional triamcinolone	Bednarek et al^34^
Trunk & extremities	Idiopathic	52-80	Acitretin	Acitretin	Current
	Idiopathic		Excision Acitretin Subcutaneous MTX	Subcutaneous MTX	Filippi et al^9^
	Nilotinib (1)		ED&C	ED&C	Crain et al^24^
	Pembrolizumab		Hydroxychloroquine	Hydroxychloroquine	Crow et al^26^
			UVB phototherapy & crude coal tar Topical clobetasol & intralesional triamcinolone	UVB phototherapy & crude coal tar Topical clobetasol & intralesional triamcinolone	
	Idiopathic		Antihistamines Topical corticosteroids Phototherapy Excision Acitretin	Excision Acitretin	Mascitti et al^30^
Face, trunk, & extremities	Nivolumab (4)	52-79	Topical clobetasol & oral acyclovir Topical halobetasol propionate Prednisone	Prednisone	Sullivan et al^8^
	Idiopathic		Isotretinoin Antihistamines Oral MTX Cyclophosphamide	None	Liu et al^15^
	Sorafenib (14)		Excision d/c sorafenib Acitretin	Excision Acitretin	Abbas et al^17^
	Idiopathic		Acitretin & topical tretinoin	Acitretin & topical tretinoin	Rotola et al^29^
	Ruxolitinib (20)		Excision	Excision	March-Rodriguez et al^32^

Table References in Supplementary Material, available via Mendeley at https://doi.org/10.17632/9kvg3s5j49.1.

*5-FU*, 5-Fluorouricil; *ED&C*, electrodessication and curettage; *MTX*, methotrexate; *PDL1*, program death ligand 1; *PDT*, photodynamic therapy; *TGF-β*, transforming growth factor beta.

Treating GEKA is clinically challenging, with no uniform approach established in past literature. Therapeutic modalities such as surgical excision or physical destruction with cautery, cryotherapy, curettage, and laser ablation have been trialed for large keratoacanthomas in GEKA.^2^ Additionally, utilization of topical therapies, including corticosteroids, 5-fluorouracil, MTX, and triamcinolone have yielded success in some cases. Other systemic therapies have included aminopterin, MTX, cyclophosphamide, γ-interferon, and α-interferon, as generalized keratoacanthoma may be resistant to initial treatment with first-line agents.^1^^,^^2^^,^^4^^,^^5^ Systemic acitretin or other oral retinoids remain first-line options for GEKA. In the last 5 years, acitretin was utilized in 12 out of 41 cases (Table I).

In this case, our patient had tried topical and non-pharmacologic treatment modalities before finally responding to low-dose acitretin. This case further supports the complicated course of GEKA, the difficulties of choosing the most beneficial treatment regimen, and the utility of acitretin in promoting disease improvement and remission.

## Conflicts of interest

None disclosed.
